# Supplementary material for: BRAFV600E Positivity-Dependent Effect of Age on Papillary Thyroid Cancer Recurrence Risk
Source: Cancers (Basel). 2023 Nov 13;15(22):5395. doi: 10.3390/cancers15225395 (PMC10670702; doi:10.3390/cancers15225395)
Supplement: Supplementary file 1 [file cancers-15-05395-s001.zip › cancers-2691994-supplementary.pdf]

Supplementary Table S1. Primer/Probe ID and Sequences of BRAF gene mutation (codon600).

**BRAF (codon 600)**		
**GenBank ID**	**Primer/Probe ID**	**Primer/Probe Sequences (5' to 3')**
NM_004333	Forward	TCATAATGCTTGCTCTGATAGGA
	Reverse	GGCCAAAAATTTAATCAGTGGA
	FL Probe	AGCTACAGTGAAATCTCGATGGAG-FL
	LC Probe	LCRed705-GGTCCCATCAGTTTGAACAGTTGTCTGGA-P
